# Effect of Curcumin Against *Proteus mirabilis* During Crystallization of Struvite from Artificial Urine

**DOI:** 10.1155/2012/862794

**Published:** 2011-07-25

**Authors:** Jolanta Prywer, Agnieszka Torzewska

**Affiliations:** ^1^Institute of Physics, Technical University of Lodz, ul. Wólczańska 219, 93-005 Lodz, Poland; ^2^Department of Immunobiology of Bacteria, Institute of Microbiology, Biotechnology and Immunology, University of Lodz, ul. Banacha 12/16, 90-237 Lodz, Poland

## Abstract

We investigated the activity of curcumin against *Proteus mirabilis* and the struvite crystallization in relation to urinary stones formation. In order to evaluate an activity of curcumin we performed an *in vitro* experiment of struvite growth from artificial urine. The crystallization process was induced by *Proteus mirabilis* to mimic the real urinary tract infection, which usually leads to urinary stone formation. The results demonstrate that curcumin exhibits the effect against *Proteus mirabilis* inhibiting the activity of urease—an enzyme produced by these microorganisms. Addition of curcumin increases the induction time and decreases the efficiency of growth of struvite compared with the absence of curcumin. Interestingly, the addition of curcumin does not affect the crystal morphology and habit. In conclusion, curcumin has demonstrated its significant potential to be further investigated for its use in the case of struvite crystallization induced for the growth by *Proteus mirabilis* in relation to urinary stone formation.

## 1. Introduction

A large number of people (up to 20% of the population worldwide) are suffering from urinary stone problem [1]. The majority of stones are composed of oxalates, calcium salts, and phosphates. Among phosphates, magnesium ammonium phosphate hexahydrate (MAPH; MgNH_4_PO_4_·6H_2_O), known as struvite, is the predominant crystalline component. Struvite crystallization is related to urinary tract infections by microorganisms producing urease. They are mainly the microorganisms from species of *Proteus*, which are isolated in the case of 70% of the so-called infectious stones [2, 3]. Urine of a healthy person is undersaturated with regard to struvite formation. However, struvite stones are formed in the case of urinary tract infection by urease-producing bacteria. This happens because urease—bacterially produced enzyme—splits urea, (NH_2_)_2_CO—natural component of urine—into ammonia,NH_3_, and carbon dioxide, CO_2_, according to the following reaction [4]:

(1) $${\left(\text{N}{\text{H}}_{2}\right)}_{2}\text{CO}\overset{\text{urease}\;+\;{\text{H}}_{2}\text{O}}{\to }\text{C}{\text{O}}_{2}+2\text{N}{\text{H}}_{3}$$

This in turn raises the pH of the urine. Increasing urinary pH leads to the elevation of the concentration of NH_4_^+^, CO_3_^2−^ and PO_4_^3−^ ions. These ions together with the ions of magnesium Mg^2+^ present in the urine lead to the crystallization of struvite, according to the following reaction [5]:

(2) $$\text{M}{\text{g}}^{2+}+\text{N}{\text{H}}_{4}^{+}+\text{P}{\text{O}}_{4}^{3-}+6{\text{H}}_{2}\text{O}\overset{\text{pH}\;\geq \;7.2}{\to }\text{MgN}{\text{H}}_{4}\text{P}{\text{O}}_{4}\cdot 6{\text{H}}_{2}\text{O}$$

The precipitation of struvite occurs for pH ≥ 7.2. The value of pH is strongly correlated with supersaturation [6]. 

Struvite formation is usually associated with carbonate apatite, Ca_10_(PO_4_)_6_CO_3_, precipitation because ions of calcium Ca^2+^ are present in urine. The precipitation of carbonate apatite runs according to the following reaction [5]:

(3) $$\text{C}{\text{O}}_{3}^{2-}+10\text{C}{\text{a}}^{2+}+6\text{P}{\text{O}}_{4}^{3-}\overset{\text{pH}\geq 6.8}{\to }\text{C}{\text{a}}_{10}{\left(\text{P}{\text{O}}_{4}\right)}_{6}\text{C}{\text{O}}_{3}$$

This component does not form crystals of defined morphology typical to struvite, it forms an amorphous precipitate. 

Struvite together with small amount of carbonate apatite (CA) forms the so-called infectious or struvite stones, which are related to urinary tract infection. Struvite stone may grow very rapidly and may involve the entire renal pelvis and calyces, which may lead to the blockage of the urinary tract and other serious medical problems including losing a kidney [7, 8]. Therefore, a number of various drugs have been developed to control this disease. Currently, a long-term antibiotic treatment is advised in the case of infectious stones [9]. Such a treatment should prevent also the recurrence and regrowth of stone after treatment. However, long-term systematic antibiotic therapy may be associated with development of many adverse effects such as gastrointestinal problems or the emergence of bacterial resistance. Therefore, several phytotherapeutic compounds have recently been investigated to treat or prevent bacterial infections which may lead to urinary stones [10, 11]. We focus our attention on curcumin. 

**Figure 1:**
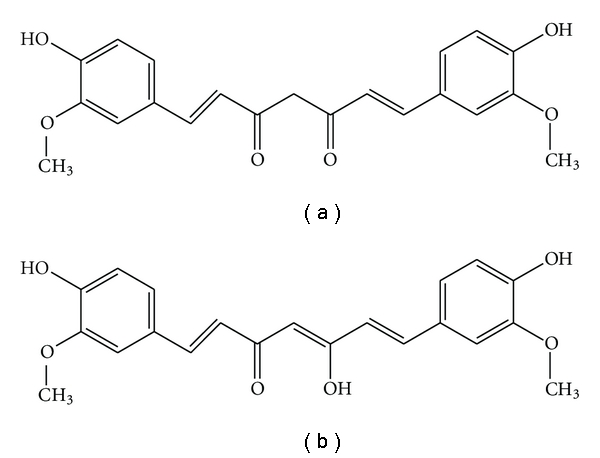
The configuration of keto form (a) and the most energetically stable enol form (b) of curcumin.

Curcumin (diferuloylmethane, chemical formula: C_21_H_20_O_6_) is a yellow-orange pigment extracted from the roots of turmeric (*Curcuma longa*). It usually exists in two tautomeric forms: keto and enol–both these forms are presented in Figure 1. The enol form is more energetically stable [12]. Curcumin has attracted attention because of its various biological activities [13]. Modern interest in turmeric began in 1970s when it was found that turmeric possesses antiinflammatory properties [14]. The significance of turmeric in medicine has changed since the antioxidant properties of curcumin were discovered [15, 16]. Therefore, curcumin is widely studied and it is found that curcumin possesses also antitumour [17, 18], antibacterial [19, 20], antifungal [21, 22] and antiviral [23, 24] properties. These studies suggest that curcumin is a potent agent, which may be applied in various pharmacological areas. Additionally, curcumin does not exhibit toxicity to either animal or humans even at high doses. Pharmacologically curcumin is safe even at a dose of 8–10 g/day [25–27].

In a previous paper [28] we have reported the characterization of the structure of struvite crystals and some results on the effect of curcumin on its biomineralization in artificial urine. In the present study, we evaluate the properties associated with curcumin with a view to extend the use of curcumin in the case of struvite crystallization induced by *Proteus mirabilis *in relation to urinary stone formation. For this purpose, we performed *in vitro* experiments of struvite crystals mineralization from artificial urine in the presence of *P. mirabilis*. To our best knowledge, our study is the first study aiming to evaluate the activity of curcumin in the course of urinary stone formation.

## 2. Materials and Methods

Curcumin (C_21_H_20_O_6_, CAS number: 458-37-7) was purchased from Sigma and is a polyphenolic compound extracted from *Curcuma longa* which belongs to *Zingiberaceae* family. 

*Proteus mirabilis* strain was isolated from human kidney stone. Before the crystal growth experiment, bacteria were maintained on a slant of tryptic soy agar overnight at 37°C and then suspended in artificial urine with or without curcumin to the concentration of 5 × 10^5^ CFU per mL. 

The artificial urine used during crystal growth experiments was made from the following components [29] (g/L): CaCl_2_·2H_2_O, 0.651; MgCl_2_·6H_2_O, 0.651; NaCl,4.6; Na_2_SO_4_, 2.3; KH_2_PO_4_, 2.8; KCl, 1.6; NH_4_Cl, 1.0; urea, 25.0; creatine, 1.1; tryptic soy broth (TSB), 10.0. The content of the mineral components in such artificial urine corresponds to mean concentration found in 24-hour period in normal human urine. The crystallization occurs after addition of the suspension of bacteria and incubation at 37°C. The crystallization process occurred at conditions emulating the natural conditions existing at human body during the infection by *Proteus sp*. During all the growth experiments, crystal samples were taken at regular intervals and observed by phase-contrast microscopy (Nikon Eclipse TE2000-S). The pH of the solution of artificial urine was screened along the experiments using digital pH-meter (Elmetron CP-215). Using this equipment the pH measurements are done with accuracy of 0.01. In the paper the average results with accuracy of 0.1 are given. The initial pH of artificial urine was adjusted to a value of 5.8. The absorbance was measured with the spectrophotometer Ultrospec 2000 (Pharmacia Biotech) at 630-nanometer wavelength. 

Before the crystal growth experiment bacterial urease activity and viability of bacteria in the presence of curcumin were established. *P. mirabilis* suspension (5 × 10^5^ CFU/mL) was added to appropriate culture medium containing series of curcumin dilution in wells of microtiter plates. To test urease activity the compound was diluted in Christensen medium with urea and a phenol red as a pH-indicator while antimicrobial activity was determined in TSB. The curcumin concentration tested was of the range 0.1 to 10 mM, and its biological activity was assessed after 24 h incubation at 37°C. After incubation, curcumin concentration inhibiting activity of urease (no change of Christensen medium color) was determined. Additionally, bacterial growth in TSB testifying the bacterial viability was observed.

## 3. Results and Discussions

Before the crystal growth sets of experiments we have examined concentrations of curcumin in the range from 0.1 to 10 mM. The experiment performed with the use of the method described in previous section has demonstrated that none of the studied concentrations of curcumin acts as bactericide. The performed experiments also show that curcumin at a concentration lower than 1 mM does not influence the activity of urease. The concentration equal to 1 mM was the smallest one for which curcumin acts as inhibitor in relation to urease activity. For concentration higher than 1 mM the inhibiting influence of curcumin on activity of urease was still observed, however, more and more greater problems with solubility of curcumin appeared as described later in this section. The results of crystal growth presented below were obtained for the concentration of curcumin equal to 1 mM.

**Figure 2:**
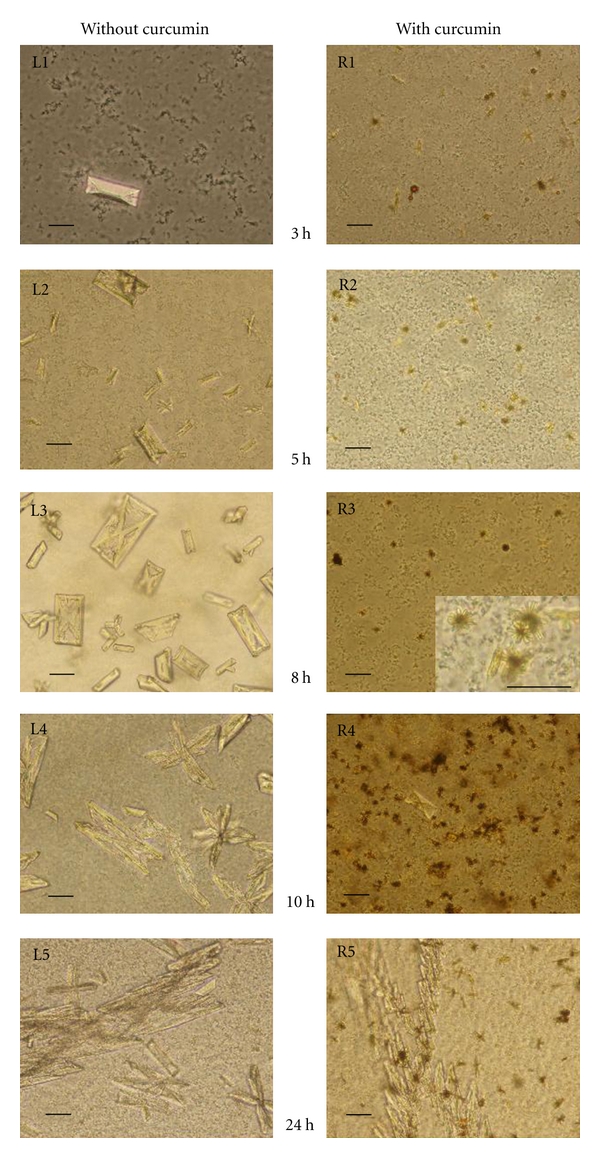
Struvite crystals grown from artificial urine infected with *Proteus mirabilis* in the absence (left column) and in the presence of curcumin (right column). Scale bar in all pictures: 20 *μ*m.

The results of our crystal growth sets of experiments are presented in Figure 2. The columns, left and right, present the course of growth process of struvite crystals grown from artificial urine in time, in the absence and in the presence of curcumin, respectively. 

**Figure 3:**
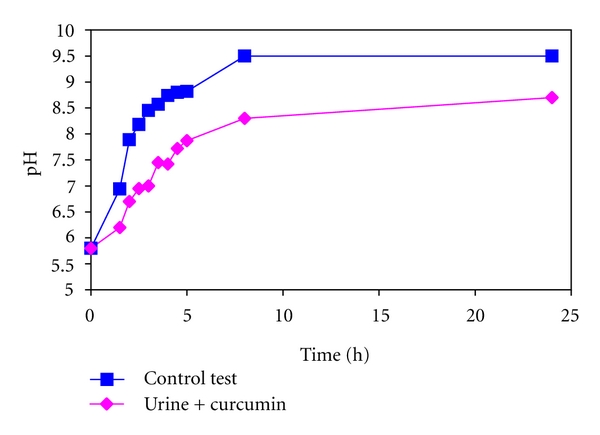
Kinetics of pH of the infected artificial urine without (control test) and with curcumin.

First, let us analyse the growth process in the absence of curcumin. In this case, first single struvite crystals were observed approximately at the third hour of the experiment, for pH higher than 7.2 (Figure 2, panel L1). With time and with increase in pH the amount of crystals is greater (Figure 2, panel L2). The most basic crystal morphology is typical hemimorphic morphology of coffin-lid shape. The hemimorphism and struvite morphology is analysed in detail in [30]. The size of the largest crystals is about 55–60 *μ*m along *b*-axis. When pH increases further the habits of single crystals remain the same, but the crystals very frequently form twins. First of all the crystals show contact twinning (Figure 2, panel L3), then contact twining turns into penetration twinning (Figure 2, panel L3 and L4). For the highest value of pH dendritic branches and dendrites appear (Figure 2, panel L5). The highest value of pH equal to 9.5 was achieved after 8 h of incubation. The pH level is correlated with the bacteria vitality, pH higher than 8 acts as bactericide. The evolution of pH of urine in time is presented in Figure 3.

In the case of addition of curcumin our experiment runs differently. First of all, the solubility of curcumin in the solution of artificial urine is relatively low. Therefore, we have initially observed unsolvable particles of curcumin which then turn into “stellar” aggregates (Figure 2, panel R1, R2, R3, and inset in panel R3). Besides, individual struvite crystals appear later (Figure 2, panel R4) compared with the absence of curcumin. Additionally, the size and number of struvite crystals decrease with addition of curcumin (Figure 2, panel R4). The largest crystals are of size 25 *μ*m along *b*-axis. The majority of formed single crystals are typical coffin-lid shape, the same as in the case of absence of curcumin. Therefore, we conclude that curcumin does not affect the crystal morphology and habit. With time and with further increase in pH very large X-shaped dendrites appear (Figure 2, panel R5). From this it follows that the growth process is strongly modified when curcumin is applied.

Figure 3 illustrates an example of pH profile with and without curcumin obtained at one of the set of experiments. One may notice that this profile is modified in the presence of curcumin. In particular, in the presence of curcumin pH of the urine increases much more slowly compared with the absence of curcumin. The pH equal to 7.2, which is required for struvite precipitation, is achieved after 4 hours of incubation, while in the case of absence of curcumin after 2.5 hours of incubation. Consequently, struvite crystals appear much later in the presence of curcumin compared with the absence of curcumin. Slower increase in pH value may be caused by two reasons. Firstly, curcumin may act as bactericide. Secondly, curcumin may inhibit the activity of urease, not influencing the bacterium's vitality. In our experiment the second case arises: curcumin inhibits the activity of urease. None of the examined concentrations of curcumin acts as bactericide. As a consequence of inhibiting influence of curcumin on urease activity, the pH value increases much more slowly in the presence of curcumin. As the pH of the solution is correlated with its supersaturation we may conclude that curcumin preserves the urine undersaturated with respect to struvite formation by inhibiting the activity of urease. The urine is undersaturated for much longer time compared with the absence of curcumin.

It is worth noting that the urease activity was estimated using well-known and widely accepted method of direct measurement of the change in pH [31–34].

**Figure 4:**
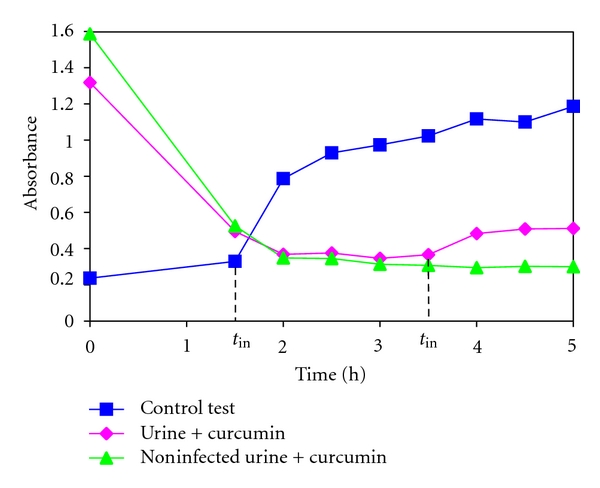
Absorbance of infected artificial urine versus time without (control test) and with curcumin and absorbance of noninfected urine with curcumin versus time.

Late appearance of struvite crystals in the presence of curcumin compared with the control test (without curcumin) may suggest also that the nucleation occurs later in the presence of curcumin. In order to verify this idea, we measured the turbidity of the artificial urine in the presence and absence of curcumin during the experiments in order to estimate the induction time, *t*_in_ (time to induce formation of detectable crystals), which characterizes the nucleation process. The turbidity of a suspension with bacteria is commonly measured as the absorbance of light of a defined wavelength [35, 36]. In our experiments the absorbance was measured with spectrophotometer Ultrospec 2000 (Pharmacia Biotech) at 630-nanometer wavelength.  Figure 4 presents the absorbance-time graph in the case of presence and absence of curcumin. This graph shows that in the case of control test (without curcumin) the absorbance initially reaches the values in the range 0.2-0.3 in absorbance unit. These values of absorbance correspond to absorbance of the solution of artificial urine with *P. mirabilis* bacteria of the concentration approximately equal to 5 × 10^5^ CFU mL^−1^. During the first 3 hours of the experiment the concentration of bacteria increases twice and then it stays constant up to the end of the experiment. However, as the pH higher than 8 acts as bactericide the number of alive bacteria systematically decreases. These changes in number of living bacteria have no significant influence on the absorbance values. On the basis of Figure 4 one can see that the initially reached value of absorbance then significantly increases. We consider that the appearance of crystals is detected when there is a sudden increase in the absorbance. The time related with this sudden increase is considered as induction time *t*_in_. In the case presented in Figure 4, *t*_in_ is equal to 1.5 h. However, Figure 4 presents an exemplifying result which is representative for all sets of experiments. In the sets of performed experiments the induction time does not exceed 2 hours in the case of absence of curcumin. Such a variation in induction time is admissible taking into account that the artificial urine with *P. mirabilis* is a complex biological environment. 

In the case of addition of curcumin it is found that the absorbance initially has the highest value which decreases until it reaches a constant value approximately equal to 0.4. Then, the absorbance increases with time. The initially high value of absorbance is correlated with weak solubility of curcumin in artificial urine. As mentioned above, just after addition of curcumin into solution of artificial urine many unsolvable particles of curcumin were observed. These unsolvable particles give initially high value of absorbance. With time these particles combine and form “stellar” aggregates inducing the decrease of absorbance to a constant value. In the case of noninfected urine this constant value remains the same up to the end of the experiment (Figure 4). However, in the case of infected urine, after achieving this constant value, an increase in absorbance occurs. This increase in absorbance after achieving this constant value is related with the appearance of nuclei of detectable size, that is, with induction time. It is clear that the induction time with the presence of curcumin is much longer compared with the control test (without curcumin). Therefore, the nucleation in the presence of curcumin occurs later compared with the control test. Moreover, Figure 4 shows that with addition of curcumin, lower absorbance intensity is measured. This means that in the presence of curcumin less detectable crystals are formed compared with control test (without curcumin). Based on the classical homogeneous nucleation theory [37], these results suggest that the addition of curcumin shifts the Gibbs free energy and critical nuclei radius to larger values. Larger critical nuclei radius means that the system needs larger nuclei to precipitate stable crystals, therefore the induction time is longer. The longer induction time in the case of addition of curcumin suggests also that the system needs higher energy to overcome the barrier to precipitate crystals. 

In our experiments both the homogeneous nucleation and heterogeneous nucleation are possible. However, it seems that the heterogeneous nucleation plays a key role because this nucleation may be easily initiated on the bacterial cells, which play the role of active sites. In the case of the addition of curcumin there are unsolvable particles of curcumin in the solution which may also serve as active sites for heterogeneous nucleation. Therefore, in the case of addition of curcumin there are much more places where the heterogeneous nucleation may be initiated. It is known that the increase in the number of such places is related to the decrease in crystal size. Therefore, the crystals are smaller compared with the crystals which grow in the case of absence of curcumin in the same conditions. Additionally, with time and with the increase of pH some of the small crystals form aggregates rather than grow into large crystals. Consequently, struvite crystals which grow with the presence of curcumin are smaller and the number of crystals is less compared with the absence of curcumin. These findings may suggest that, at this stage, struvite crystals may be easily removed from urinary tract with urine. However, with further increase in pH very large X-shaped dendrites appear in the presence of curcumin. We suggest that the formation of these large dendrites may be associated with large number of 3D nuclei and their aggregation in the presence of curcumin. These large dendrites may easily be retained in the urinary tract and consequently, at this stage, may stimulate the crystallization process and stone formation.

The relative standard error (RSE) of the absorbance measurements does not exceed 4% for control test. In the case of infected urine with curcumin, for the first stages of experiment (up to approximately 2 hours of the experiment) in all sets of experiments the RSE of absorbance is on the level of 10%. Afterward, beginning from 2 hours of experiments, RSE is lower and does not exceed 5%. Similar situation is in the case of noninfected urine with curcumin. From this data it follows that the standard deviation and resulting from it RSE is influenced mainly by curcumin. In particular, the higher values of RSE at the first stages of experiments testify about the considerable influence of unsolvable particles of curcumin on absorbance. With time the system attains an equilibrium state and the RSE of absorbance is lower. All these RSE values are admissible in this dynamic and complex biological system. Independently on the RSE of absorbance the induction times change in the range from 1.5 to 2 hours in the case of control test and from 3.5 to 4 hours in the case of infected urine with curcumin. 

Weak solubility of curcumin is known and described in the literature (e.g., [25]) and may be a limiting factor of its therapeutic utility. Therefore, many attempts are currently made to develop methods to improve its solubility.

## 4. Possible Mechanism of Urease Inhibition by Curcumin

Ureases have been isolated from a wide variety of organisms including plants, fungi, and bacteria. However, major structural differences have been observed between the ureases derived from plants and microorganisms. The urease of *Proteus mirabilis* is a nickel-containing enzyme and is composed of three subunit polypeptides [38]. More exactly speaking, the urease active site is found to have pseudooctahedral, paramagnetic, and binuclear nickel ions. The urease-Ni ions play the key role in the mechanism of urea degradation [39]. Urease inhibitors interact with it and block its activity by inhibiting the urea hydrolysis to ammonia and carbon dioxide. Urease inhibitors can be divided into two major categories: (i) active-site directed mode; (ii) mechanism-based directed mode. The active-site directed inhibitors show close structural similarity with urea—the enzyme's substrate. Mechanism-based directed inhibitors interfere with the enzyme's catalysis mechanism leading to its inhibition or inactivation [36]. 

In our opinion, curcumin appears to be mechanism-based urease inhibitor. The chemical structure of curcumin consists of two *o*-methoxy phenols attached symmetrically through *α*, *β*-unsaturated *β*-diketone linker, which also induces keto-enol tautomerism [25, 40], so it is a natural polyphenol. It is possible that this compound inhibits urease activity through a chelate interaction, which binds to the urease active site; thereby curcumin makes a stable complex with urease and in turn the inhibition of urease activity. The diketone moiety of curcumin possesses chelating abilities towards transition metals, to which nickel belongs also. The chelation of curcumin towards transition metals such as iron and copper has been found to be useful in the treatment of Alzheimer's disease [41].

The interaction between urease and curcumin may result also from the electrostatic stabilization. Our results may suggest that we deal with a form of mixed inhibition, where the binding of the inhibitor to the urease active sites reduces its activity but does not affect the binding of urea. We suppose such a mechanism because the extent of the inhibition of curcumin depends strongly on its concentration. 

We consider our suggestions as stimulation of further work to study the mechanism of urease inhibition by curcumin. In order to establish the details of such a mechanism, the structural changes occurring in urease upon its interaction with curcumin should be examined. Understanding the mechanism of curcumin interaction with urease of *Proteus mirabilis* should be the next step towards the development of future therapeutic agents. The results described in this study may be important preliminary step to achieve the goal.

## 5. Conclusions

The results of our study demonstrate that curcumin inhibits the activity of urease-bacterially produced enzyme, which is driving force of struvite crystallization. Therefore, the pH level of urine increases much more slowly in the presence of curcumin compared with the absence of curcumin. For this reason urine is undersaturated with respect to struvite formation for longer time compared with the control test (without curcumin). Increase in pH leads finally to crystallization of struvite. The morphology of growing crystals is the same as in the case of the absence of curcumin. However, the crystals are smaller and the number of crystals is less compared with the control test (without curcumin). Therefore at this stage the crystals may be easily removed from urinary tract with urine. With further increase in pH very large X-shaped dendrites grow, larger than those in the case of the absence of curcumin. Such large dendrites may be retained in urinary tract and, in consequence, may promote the formation of struvite. In conclusion, the results demonstrate that curcumin has interesting and promising properties, which combined with pharmacological safety, render curcumin an attractive agent to explore further.
